# True gene-targeting events by CRISPR/Cas-induced DSB repair of the PPO locus with an ectopically integrated repair template

**DOI:** 10.1038/s41598-018-21697-z

**Published:** 2018-02-20

**Authors:** Sylvia de Pater, Bart J. P. M. Klemann, Paul J. J. Hooykaas

**Affiliations:** 1 Department of Molecular and Developmental Genetics, Institute of Biology, Leiden University, Leiden, 2333 BE The Netherlands; 2 Present Address: Dümmen Orange, De Lier, 2678 PS The Netherlands

## Abstract

In recent years, several tools have become available for improved gene-targeting (GT) in plants. DNA breaks at specific sites activate local DNA repair and recombination, including recombination with ectopic sequences leading to GT. Large-scale transformation with the repair template can be avoided by pre-insertion of the repair template in the genome and liberation by sequence-specific nucleases (in planta GT procedure). Here, we tested whether release of the repair template was required for GT. Plants were transformed with constructs encoding a CRISPR/Cas nuclease with a recognition site in the endogenous *PPO* gene and a repair template harboring a 5′ truncated *PPO* gene with two amino acid substitutions rendering the enzyme insensitive to the herbicide butafenacil. Selection resulted in so-called true GT events, repaired via homologous recombination at both ends of the gene and transmitted to the next generation. As the template was surrounded by geminiviral LIR sequences, we also tested whether replication of the template could be induced by crossing-in an integrated geminivirus *REP* gene. However, we could not find evidence for repair template replication by REP and we obtained similar numbers of GT events in these plants. Thus, GT is possible without any further processing of the pre-inserted repair template.

## Introduction

One of the tools that is of great value for fundamental and applied research is the precise modification of DNA sequences. This so-called gene-targeting (GT) technique utilizes homologous recombination (HR) to precisely exchange genomic sequences^1^. GT is a very rare event in higher plants and in one of the first reports in plants frequencies as low as 10^4^ were found^2^. In the past decades it was found that by introduction of a DNA break at the site of intended recombination the low frequency of GT in plants can be enhanced^3–9^. Targeted double strand breaks (DSBs) can be generated using sequence-specific nucleases, including meganucleases, zinc finger nucleases (ZFNs), TAL effector nucleases (TALENs) and CRISPR/Cas nucleases (for ‘the clustered regularly interspaced short palindromic repeats’ and ‘CRISPR-associated’)^10^.

The most easy-to-use and effective type of sequence-specific nuclease for DSB induction is based on the CRISPR/Cas9 system^11^, derived from the adaptive immune system of *Streptococcus pyogenes*. This gene editing tool consists of a Cas9 protein which has two nuclease domains each cleaving one of the DNA strands and a two-RNA structure, that has been engineered into a single guide RNA (sgRNA)^11^. The specificity resides in a 20 nt sequence of the sgRNA. When no repair template is available or HR repair is not active, the DSB will be repaired via end-joining, which may result in deletions and insertions. The frequency of these so-called footprints can be used as measure for the activity of the nuclease. Nuclease-mediated DSB induction has been used to create targeted mutations in various organisms including plants (for reviews see: refs^10,12–16^). The CRISPR/Cas9 system has been applied to a variety of plant species for mutagenesis^17–22^ and GT^9,23,24^.

When a DSB is repaired via HR, the sister chromatid or homologous chromosome is preferably used for repair, but with low frequency also homologous sequences on the homologous chromosome may be used. A repair template can also be supplied exogenously by transformation^2^. However, as only a limited number of cells will be transformed, in a newer procedure, called *in planta* GT, the repair template was pre-inserted (as T-DNA) in the genome and released in plants containing the construct in all of its cells where sequence-specific nucleases are expressed^24,25^. However, the number of repair templates available for *in planta* GT is limited to one per cell if only one copy of the repair template had been pre-inserted in the genome. As an alternative, delivery of repair templates using viruses may enhance the frequency of GT events. The geminivirus bean yellow dwarf virus (BeYDV) was used for delivery of nuclease genes and repair templates in tobacco, tomato and wheat^26–28^. In this way, replication of the repair template was observed to enhance the GT frequency^26^.

Previously, we showed that DSB-induction in the natural *PPO* gene using ZFNs enhanced GT^5^. In these experiments, homologous recombination between the endogenous *PPO* gene and the repair construct occurred directly after delivery of the repair template as T-DNA by *Agrobacterium* floral dip transformation. A limited number of cells are being transformed with this method and only in a small fraction of these transformed cells the repair template will be integrated via homologous recombination. In this article, we tested GT at the *PPO* locus by combining local DSB induction by CRISPR/Cas with a pre-inserted repair template, the principle of *in planta* GT. We found that GT is possible even without release of the repair template. The true GT events were stable and heritable.

## Results

### Parental lines for in planta GT

**Figure 1:**
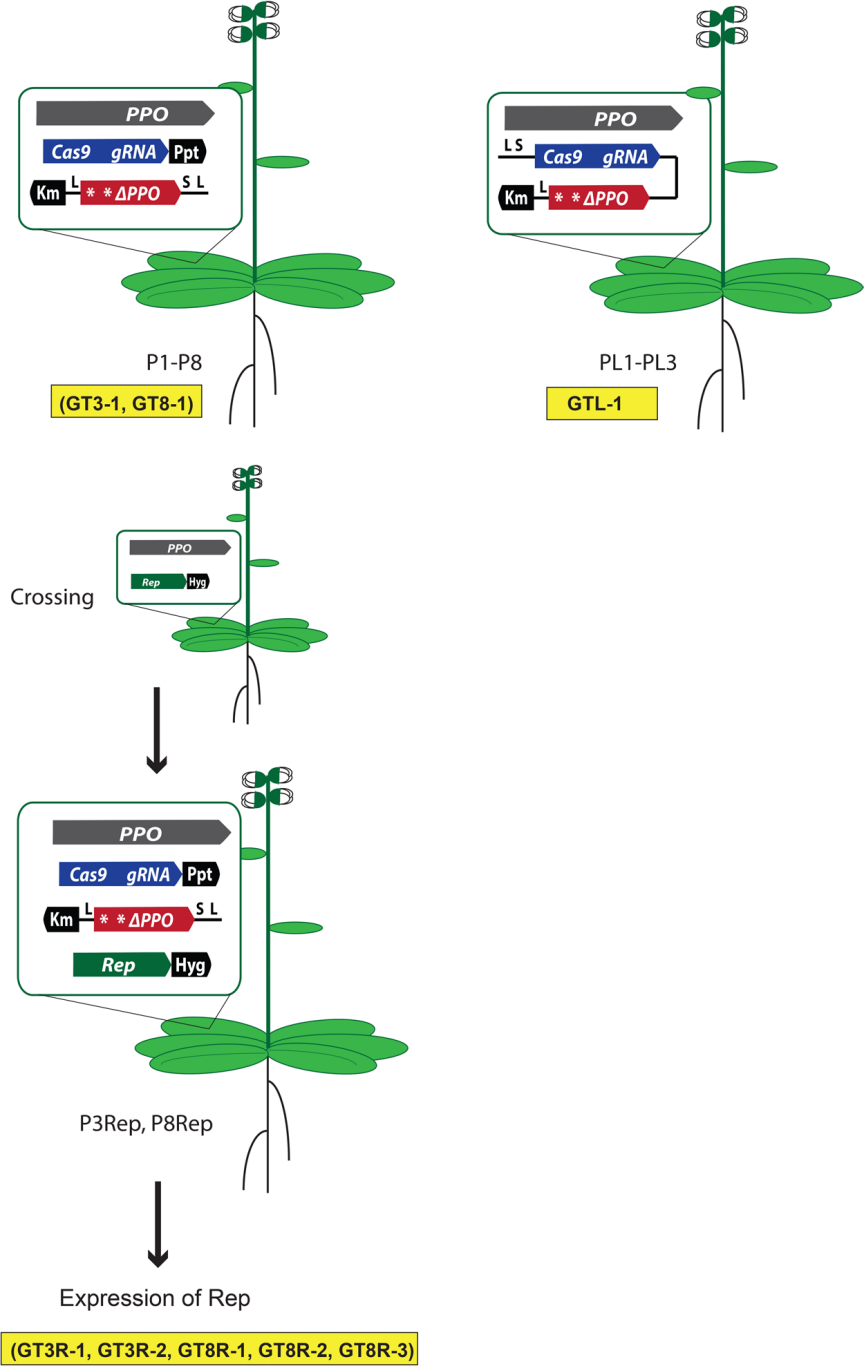
Outline of the experimental approach. Plants were transformed with the LSL-PPO repair template (km^R^) and the Cas9-PPO nuclease (ppt^R^) (P1-P8) or with Cas9-PPO and LSL-PPO on one T-DNA (km^R^) (PL1-PL3). P2, P3, P4, P5 and P8 plants were crossed with plants transformed with 35S-REP (hyg^R^). Progeny was selected on kanamycin, phosphinothricin and hygromycin for the presence of all three T-DNA constructs (P2Rep, P3Rep, P4Rep, P5Rep and P8Rep). Seed from P3, P8, PL1-PL3 (Cas9-PPO and LSL-PPO), P2Rep, P3Rep, P4Rep, P5Rep, P8Rep (Cas9-PPO, LSL-PPO and Rep), were germinated on butafenacil resulting in 8 GT plants (highlighted in yellow).

Our strategy for *in planta* GT employed a pre-inserted CRISPR/Cas nuclease gene for DSB induction at the genomic target site and a pre-inserted repair template surrounded by BeYDV geminivirus large and short intergenic regions (LIR and SIR). Plants with these pre-inserted constructs were crossed with plants containing the geminiviral replication initiation (Rep) gene. In the F1 plant cells the REP protein may act on the LIR and SIR sequences leading to rolling circle amplification of the repair template. GT events were selected in the next generation (Fig. 1).

The *protoporphyrinogen oxidase* (*PPO*) gene, for which an efficient selection system for GT has been developed^29^, was used as target. The *PPO* gene is involved in chlorophyll and heme synthesis. The encoded enzyme can be inhibited by the herbicide butafenacil, which leads to rapid plant death due to formation of reactive oxygen. Two specific amino acid changes render this enzyme insensitive to the herbicide, which can be exploited for selection of GT events by the introduction of these mutations in the *PPO* gene via homology directed repair (HDR).

**Figure 2:**
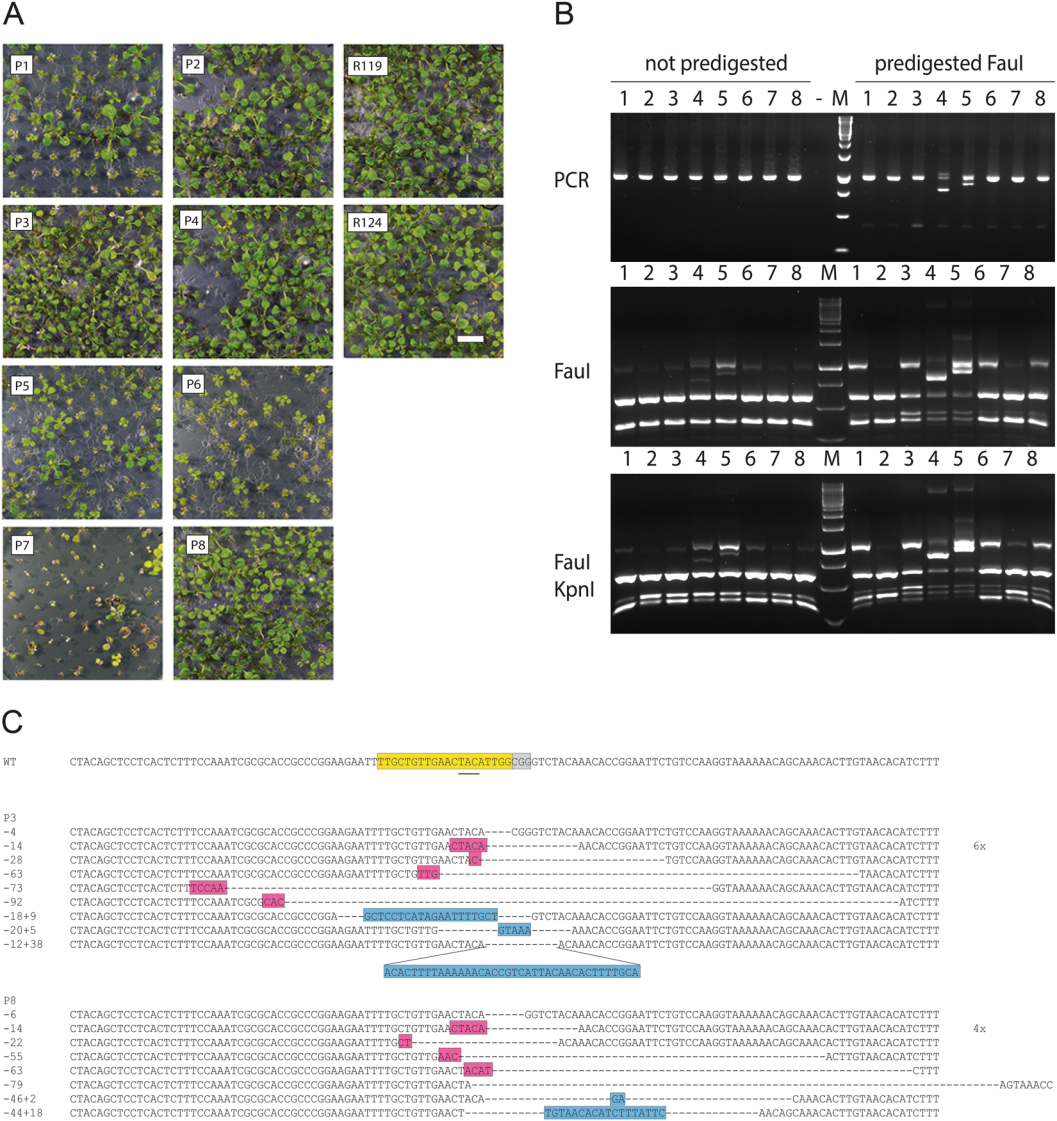
Analysis of parental lines. (**A**) T2 transformants containing the LSL-PPO repair template and the Cas9-PPO nuclease gene were germinated on kanamycin and phosphinothricin (P1-P8) and the transformants containing the 35S-REP gene on hygromycin (R119 and R124). The size bar is 1 cm. (**B**) Footprint analysis of plant lines P1-P8. Genomic DNA was pre-digested with *Fau*I or not pre-digested and the *PPO* target site was amplified and an aliquot was separated on a 1.5% agarose gel. The PCR products were digested with *Fau*I or a combination of *Fau*I and *Kpn*I and separated on 1.5% agarose gels. A negative control not containing template DNA (−) was included in the PCR reaction. M is the 1 kb DNA marker. The full-size gels are presented in Fig. S4. (**C**) Sequences of cloned target sites of P3 and P8. The sgRNA sequence is in yellow, PAM sequence in grey, insertions are in blue. Deletions are indicated by dashes and repair via microhomology in pink.

For DSB induction in the *PPO* gene a Cas9-gRNA nuclease (Cas9-PPO) was previously constructed by us^30^. The sgRNA was designed to overlap the TAC sequence that is mutated to ATG in the repair template, expecting the repair template to be resistant to the nuclease. As a repair template, the 5′ truncated *PPO* gene containing the two mutations for butafenacil resistance (S305L and Y426M) and a *Kpn*I site at position E445/A446 (pSDM3900)^5^ was cloned between the geminivirus LIR and SIR sequences (LSL-PPO). Eight plant lines were generated containing the Cas9-PPO nuclease gene and the LSL-PPO repair template by floral dip transformation with a mixture of two *Agrobacterium* strains. Several of these lines (P1, P5, P6, P7) showed growth deficiencies (Fig. 2A), indicating the presence of active Cas9-PPO, since disruption of both alleles of the *PPO* gene will be lethal. The eight plant lines (P1-P8) were further analyzed for the activity of the Cas9-PPO nuclease.

Two-week-old Cas9-PPO/LSL-PPO T2 seedlings were germinated on phosphinothricin (for selection of the T-DNA with the Cas9-PPO nuclease gene) and kanamycin (for selection of the T-DNA with the LSL-PPO repair template), and mixtures of ten seedlings were used for DNA isolation and subsequent footprint analysis. PCR amplified Cas9-PPO target sites, using primers recognizing sequences in the genomic *PPO* gene as well as in the LSL-PPO repair template, were digested with the restriction enzyme *Fau*I, which is located close to the predicted site of DSB induction. Target sites containing mutations destroying this *Fau*I site will be easily detectable as *Fau*I resistant PCR products (Fig. 2B). In order to enrich for footprints, the genomic DNA was also predigested with *Fau*I prior to PCR amplification. In six plant lines, resistant bands were detected using predigested DNA, some of which could also be observed without predigestion. Resistant bands of two lines (P3 and P8) were cloned and sequenced (Fig. 2C). Small deletions, in some cases accompanied by insertions were obtained. These results showed that the plant lines contained active Cas9-PPO nucleases. In order to determine whether the Cas9-PPO was specific for the genomic locus and inactive on the LSL-PPO repair template, the PCR products were digested with *Fau*I and *Kpn*I. Comparing the *Fau*I digestion pattern with the *Fau*I/*Kpn*I pattern suggested that most of the *Fau*I resistant bands were also resistant to *Kpn*I, and derived from the genomic locus. Out of 108 cloned *Fau*I resistant PCR fragments only one having a 8 bp deletion destroying the *Fau*I site showed both mutations (S305L and Y426M) and the *Kpn*I site present in the repair template. This indicated that the Cas9-PPO nuclease was much more active at the genomic locus and thus leaving the repair template mostly intact for *in planta* GT.

### GT in presence of a geminivirus *Rep* gene

Plants transformed with the 35S-REP gene (Rep119 and Rep124; Fig. 2A), containing an intact *PPO* gene, were crossed with Cas9-PPO/LSL-PPO lines P2, P3, P4, P5 and P8 (Fig. 1). F1 seeds were selected on plates containing phosphinothricin (for selection of the T-DNA with the Cas9-PPO nuclease gene), kanamycin (for selection of the T-DNA with the LSL-PPO repair template) and hygromycin (for selection of the T-DNA with the 35S-REP gene). Plants from different crossings containing all 3 T-DNAs were grown on soil and about 2 × 10^5^ T2 seeds in total were selected for GT events on butafenacil (Table S1). T3 seeds from positive plants were germinated on butafenacil for verification of butafenacil resistance.

**Figure 3:**
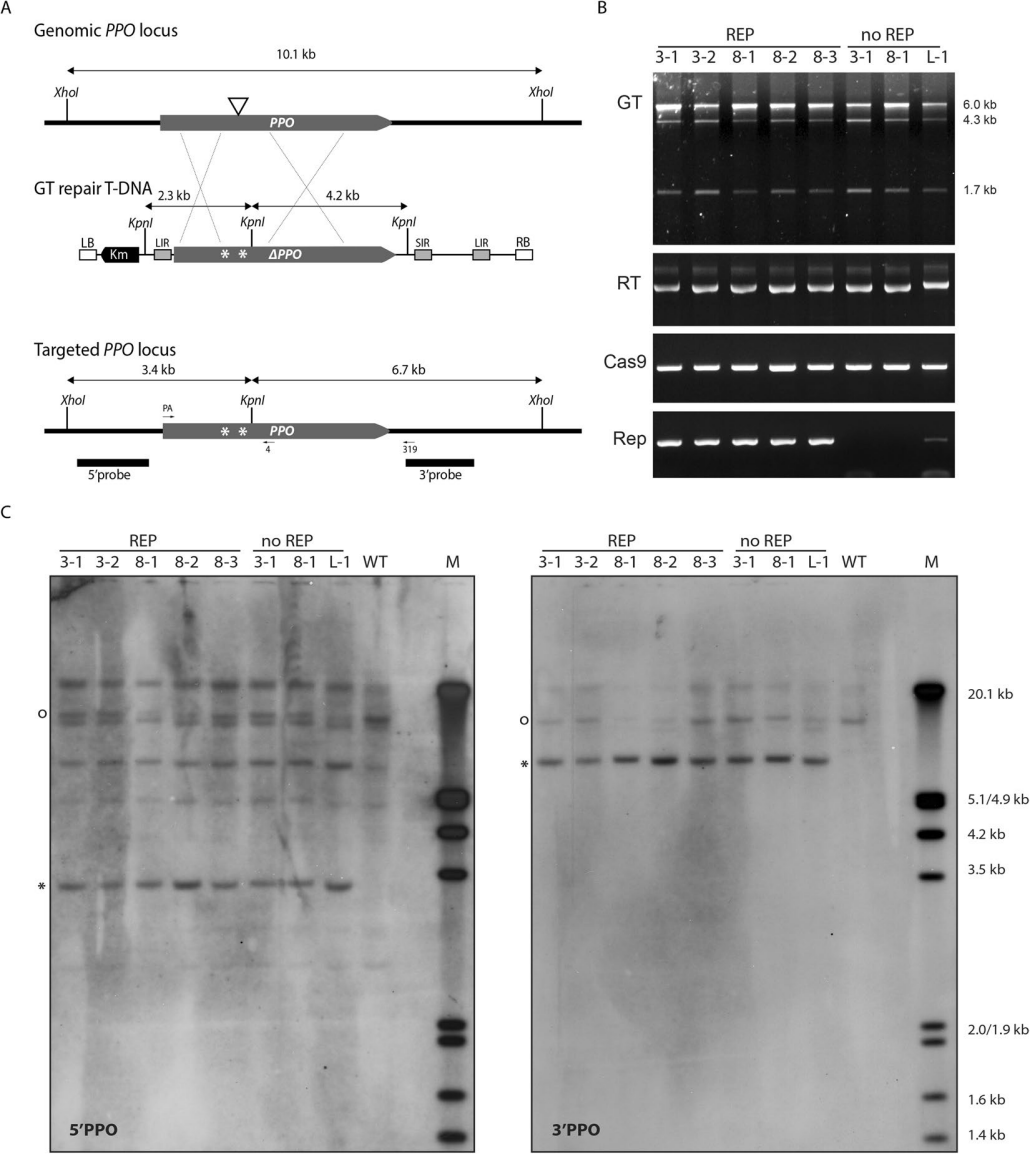
Analysis of GT events. (**A**) Structures of the wild-type genomic *PPO* locus, the LSL *in planta* repair GT construct and the targeted *PPO* locus. The coding region of the *PPO* gene is shown as a grey bar; the Cas9-PPO target site in the *PPO* gene as a triangle. The GT repair construct, missing the 5′ region of the *PPO* gene including the first 364 bps of the coding sequence, contains base pair substitutions leading to two amino acids changes (indicated by asterisks) (S305L and Y426M) causing insensitivity for the herbicide butafenacil and a *Kpn*I site for detection of GT events. The truncated *PPO* gene is surrounded by LIR and SIR sequences for rolling circle replication by the REP protein. The kanamycin gene on the GT repair construct is used for selection of transformants. Positions of primers used for PCR detection of GT events are shown. Sizes of DNA fragments expected after digestion with selected restriction enzymes are indicated. Probes used for Southern blotting are shown as black bars. (**B**) GT lines (GT3R-1, GT3R-2, GT8R-1, GT8R-2, GT8R-3, GT3-1, GT8-1 and GTL-1) were tested for the presence of GT, LSL-PPO repair template (RT), Cas9-PPO and REP by PCR. GT products were detected with primer pair PA-SP319. PCR products were digested with *Kpn*I. Sizes of bands after KpnI digestion are indicated. The full-size gels are presented in Fig. S5. (**C**) Southern blot analysis of GT lines. Ten µg DNA of wild-type plants (WT) or GT lines (GT3R-1, GT3R-2, GT8R-1, GT8R-2, GT8R-3, GT3-1, GT8-1 and GTL-1) were digested with *Kpn*I and *Xho*I, separated on 0.7% agarose gels and hybridized with a 5′*PPO* probe (left panel) or 3′*PPO* probe (right panel). The circles indicate wild-type bands (10.1 kb). Asterisks indicate bands with the expected sizes (3.4 kb and 6.7 kb) after GT by HR. Lanes M contain DIG-labelled Lambda *Eco*RI/*Hind*III marker. The sizes of the marker bands are shown. The full-size blots are presented in Fig. S6.

Five plant lines were obtained that were resistant to butafenacil, which originated from three different crossings: P3xRep119, P3xRep124 and P8xRep124. These plant lines were analyzed in detail by PCR and Southern blotting to verify the GT events. Structure of the genomic PPO locus, the LSL-PPO repair T-DNA, the recombined PPO locus, primers used for PCR and probes for Southern blotting are shown in Fig. 3A. First, these plant lines (GT3R-1, GT3R-2, GT8R-1, GT8R-2 and GT8R-3) were analyzed for GT events by PCR (Fig. 3B). The five butafenacil-resistant lines produced *Kpn*I-sensitive as well as *Kpn*I-resistant PCR products with primers PPO-PA and SP319, resulting in bands of 6.0 kb (resistant to *Kpn*I) and 4.3 kb and 1.7 kb (sensitive to *Kpn*I), indicating GT via HDR between the genomic PPO locus and the LSL-PPO repair template at the 3′ and the 5′ end of the gene, and that so-called true GT had occurred at one chromosome. This means that the plant lines were heterozygous, containing a GT allele as well as a wild-type allele. PCR analysis with primers detecting the LSL-PPO repair template, Cas9-PPO and the *Rep* gene showed that all three T-DNAs were present in these plant lines (Fig. 3B).

To exclude that the *Kpn*I sensitive PCR products were artifacts resulting from the PCR, Southern blot analysis was performed. DNA from the GT plant lines was digested with *Kpn*I and *Xho*I. The probes previously used for detecting GT events^5^ also hybridized with the LSL-PPO repair template and this resulted in many intense bands (Fig. S1). Since these bands were also observed in the parental plants (results not shown) they probably reflect multiple inserts of LSL-PPO T-DNA. The expected GT bands were not detectable on these blots. Therefore, new probes were designed detecting the GT bands and not the LSL-PPO repair template (Fig. 3A). The expected sizes of GT bands detected with the 5′ and 3′ probe are 3.4 kb and 6.7 kb respectively, indicating the correct incorporation of the *KpnI* site at the endogenous PPO locus. These bands were present on the Southern blots in all lanes with DNA from GT plants but not in the lanes with wild type DNA (Fig. 3C). In all lanes, the wild-type band of 10.1 kb was detected with both probes. The additional bands probably represented partially digested DNA or homologous sequences.

**Figure 4:**
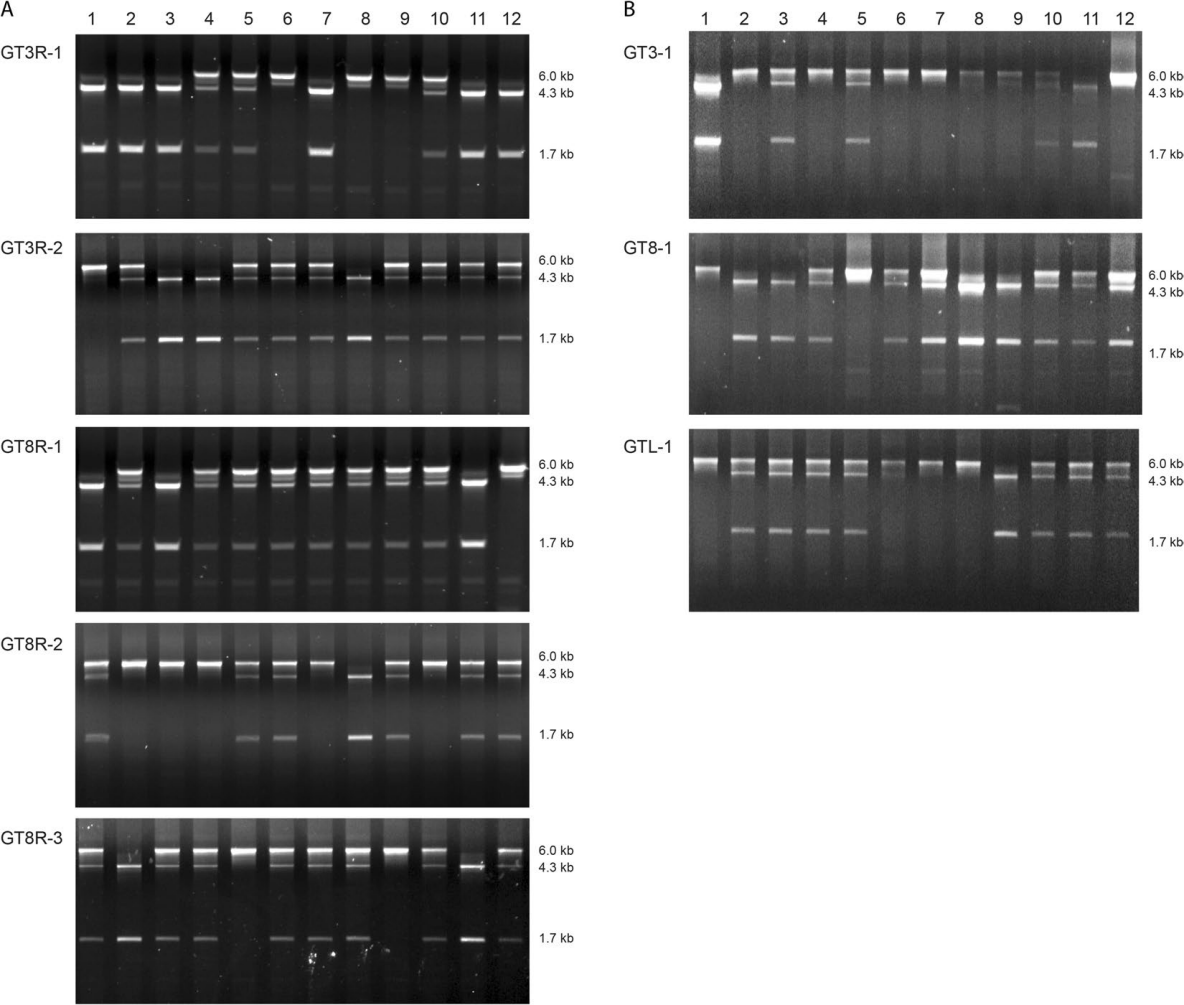
PCR analysis of progeny. (**A**) Twelve progeny plants of GT3R-1, GT3R-2, GT8R-1, GT8R-2 and GT8R-3 were tested for the presence the wild-type *PPO* locus (6.0 kb product) or GT *PPO* locus (4.3 kb and 1.7 kb products) by PCR with primers PA-SP319 and subsequent *Kpn*I digestion. The full-size gels are presented in Fig. S7. (**B**) Twelve progeny plants of GT3-1, GT8-1 and GTL-1 were tested for the presence the wild-type *PPO* locus (6.0 kb product) or GT *PPO* locus (4.3 kb and 1.7 kb products) by PCR with primers PA-SP319 and subsequent *Kpn*I digestion. The full-size gels are presented in Fig. S8.

Since the GT lines were heterozygous, progeny plants were analyzed for segregation of the GT locus. Twelve offspring plants of these lines were analyzed by PCR (Fig. 4A). They all gave homozygous, heterozygous and wild-type progeny plants, corroborating the conclusion that the lines were heterozygous true GT lines. PCR analysis of the progeny plants showed that all contained the Cas9-PPO T-DNA (Fig. S2a). Segregation was only observed for the T-DNA with the LSL-PPO repair template in GT3R-1 progeny (Fig. S2b) and for the T-DNA with the *Rep* gene in GT3R-1, GT8R-1 and GT8R-2 progeny (Fig. S2c).

**Figure 5:**
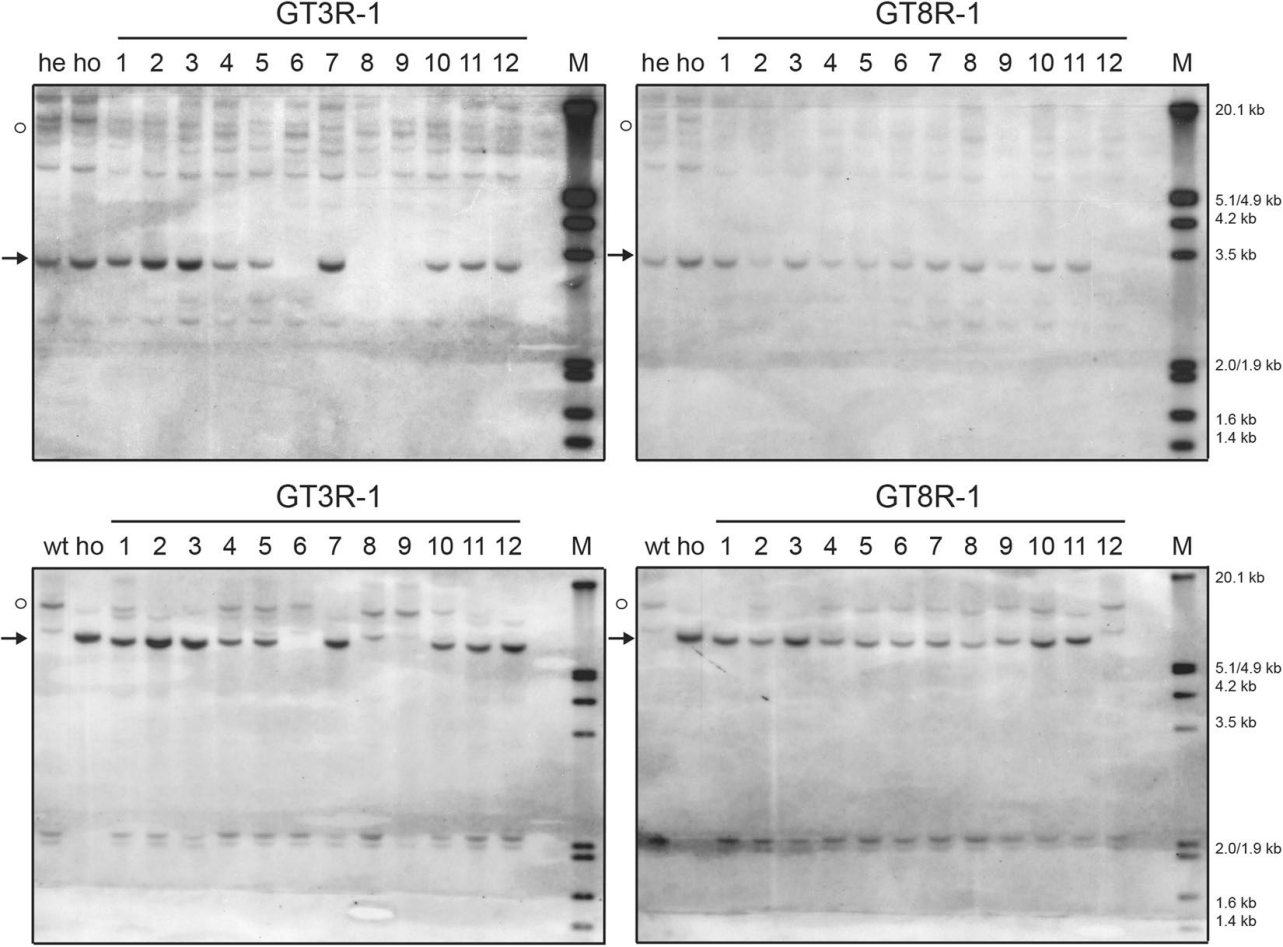
Southern blot analysis of progeny of GT3R-1 and GT8R-1. Ten µg DNA of wild-type plants (WT), control heterozygous GT plants (he), control homozygous GT plants (ho), GT3-1 and GT8-1 progeny (1-12) were digested with *Kpn*I and *Xho*I, separated on 0.7% agarose gels and hybridized with a 5′*PPO* probe (upper panels) or 3′*PPO* probe (lower panels). The dots indicate wild-type bands (10.1 kb). Arrows indicate bands with the expected sizes (3.4 kb and 6.7 kb) after GT by HR. Lanes M contain DIG-labelled Lambda *Eco*RI/*Hind*III marker. The sizes of the marker bands are shown. The full-size blots are presented in Fig. S9.

Two plant lines were analyzed in more detail. Southern blot analysis was performed on DNA from 12 progeny plants of GT3R-1 and GT8R-1 digested with *Kpn*I and *Xho*I (Fig. 5). The expected sizes of GT bands were detected with the 5′ and 3′ probe (3.4 kb and 6.7 kb respectively) in the lanes with DNA from plants that also contained GT PCR products (GT3R-1-1 to GT3R-1-5, GT3R-1-7, GT3R-1-10 to GT3R-1-12; GT8R-1-1 to GT8R-1-11) (Fig. 5). Heterozygous plants and plants without the GT locus showed bands of 10.1 kb expected for the wild-type locus (GT3R–4 to GT3R-1-6, GT3R-1-8 to GT3R-1-10; GT8R-1-2, GT8R-1-4 to GT8R-1-10, GT8R-1-12).

Sequences of PCR products with primers PPO-PA and PPO-4 of the homozygous GT3R-1 and GT8R-1 lines were identical as expected from precise homologous recombination between the endogenous *PPO* gene and the LSL-PPO repair template. In GT3R-1, mutations resulting in the amino acid substitutions S305L and Y426M and the additional *KpnI* site were present, indicating that recombination had occurred upstream of the S305L mutation. In GT8R-1, the S305L mutation was not present indicating that recombination had occurred in the region located between S305L and Y426M. The single Y426M amino acid change was sufficient to confer butafenacil resistance in GT8R-1 as was described previously^31^.

Seeds of the progeny plant lines GT3R-1 and GT8R-1 were also germinated on plates containing butafenacil. Segregation of butafenacil resistance correlated with the PCR and Southern blot results (Table S2). In conclusion, the results showed that the GT events were so-called true GT events via HDR of the endogenous PPO locus with the repair template at both the 5′ and 3′ end and that the mutations were transmitted to the next generation in a Mendelian fashion.

### GT in absence of REP

The GT3R-1 and GT8R-1 plants were analyzed for replication of the repair template by PCR using primers that would detect circularization of the region between the LIR repeats. No replication of the repair template could be detected. Subsequently, qPCR was performed to determine the number of copies of PPO sequences in these plant lines as well as in the parent and progeny lines. In GT3R-1-1, 5, 11 no LSL-PPO was detected by PCR (Fig. S2a) and indeed the amount of PPO DNA was similar as in the wild type (Fig. S3a). In the other GT3R-1 lines and the GT8R-1 lines the amount of PPO sequences resembled those in the parent P3 and P8 plants, in which the *Rep* gene is not present. Likewise, in GT3R-2 and 4 very similar amounts of PPO were found although in GT3R-1-2 Rep was present whereas in GT3R-1-4 no Rep was detected (Fig. S2c). This indicates that the presence of the *Rep* gene did not result in extra copies of PPO.

Since no replication of the repair template could be detected, we tested whether the *Rep* gene was required for GT. Seeds from Cas9-PPO/LSL-PPO lines P3 and P8 were directly selected on butafenacil (Fig. 1). In addition, seeds from plant lines (PL1-PL3) transformed with a single T-DNA containing both the Cas9-PPO gene and the LSL-PPO repair template were selected on butafenacil. Two butafenacil resistant plants (GT3-1 and GT8-1) were obtained from a total of 50.000 seeds of plant lines P3 and P8 and one butafenacil resistant plant (GTL-1) from 6000 seeds of plant line PL1 (Table S1). Molecular analysis by PCR showed that they were heterozygous for the GT locus, like the plants described above and that they contain the Cas9 and repair template T-DNAs (Fig. 3B). Southern blot analysis showed the expected sizes of GT bands of 3.4 kb and 6.7 kb at the 5′ and 3′ end respectively, in addition to the wild-type band of 10.1 kb, corroborating the PCR results (Fig. 3C). In the next generation heterozygous, homozygous and wild-type plants were obtained (Fig. 4B). PCR analyses showed that the Cas9-PPO and LSL-PPO constructs were present in all progeny plants (Fig. S2a,b). These results together showed that heritable true GT events had occurred in these plant lines even in the absence of the *Rep* gene and that replication of the repair template was not required for GT.

## Discussion

GT is strongly enhanced by a DSB at the target locus. The frequency can be further increased by the presence of a high number of repair templates. This has been achieved by transformation with repair templates surrounded by geminivirus LIR sequences on which the viral REP protein acts leading to multicopy amplification by rolling circle replication^26^. Still, transformation only reaches a limited number of target cells. Therefore, the so-called *in planta* GT procedure was proposed in which the repair template is first integrated at an ectopic site in the genome and subsequently excised to generate in all the plant cells the DNA molecule which can be used for DSB repair of the target locus. Here, we tested whether replication of a pre-inserted repair template could be achieved via a chromosomally integrated *Rep* gene acting on the LIR recognition sequences surrounding the repair template. This could theoretically lead to the presence of a high number of template copies in all the cells of the plant. In our experiments we indeed found GT events, but we obtained such GT events at similar numbers in the absence of REP. Expression of the *Rep* gene was detected in RT-qPCR assays (results not shown). However, we could not detect circularization or additional copies of the repair template. This means that the 5 GT events we obtained in plants with the Rep gene probably did not result from replication of the repair template, but resulted from recombination of the ectopically integrated repair template and the endogenous *PPO* gene, both present in the same genome, like the 3 GT events obtained in plants without the *Rep* gene. Experiments that used viral amplification for high production of proteins^32^ or to increase GT frequencies^26^ were performed after transient delivery of viral vectors. In our experiments, the sequences for viral replication were pre-inserted in the genome. Perhaps chromatin modifications prevented the replication process. It is possible that the approach that was used, might be successful at other loci.

It is remarkable that we obtained GT events by DNA recombination of the target locus with an ectopically integrated repair template. In the described *in planta* GT method, DSBs were induced both in the target locus and simultaneously at the ends of the pre-inserted repair template allowing HDR of the target locus by the excised repair template^24,25^. An advantage of the *in planta* GT method is that in each cell of the organism where the nuclease is expressed, the repair template is available for GT. No large numbers of transformants are required. In our experiments, a similar procedure was followed and we found unexpectedly that GT events were obtained even without excision of the repair template by a nuclease. Apparently, a DSB in a target locus can be repaired by a homologous sequence present elsewhere in the genome. Similar results were obtained in GT experiments in maize on an artificial locus, where excision of the donor locus was also unnecessary^33^. However, here a single DSB was induced at one end of the repair template. HDR has also been observed at low frequency with two unlinked homologous non-functional kanamycin genes^34^. In both these studies also one-sided HDR events were obtained. Here, we showed that HDR with ectopic sequences can also occur at an endogenous locus and without any DSBs at the ectopic locus. This may be the reason that we only obtained so-called true GT events with HDR at the 5′ and 3′ ends unlike the other experiments described above. An alternative explanation may be that in our system only a few base pair changes are being introduced in the endogenous gene via homologous recombination, whereas in the previous studies a gene was added, and we used Arabidopsis instead of maize or tobacco.

The most powerful site-specific nucleases that is being applied for genome editing are based on the bacterial CRISPR/Cas system^13,14^. Previously, we also observed very efficient DSB induction and mutagenesis in the *PPO* gene using the CRISPR/Cas system (Fig. 2) with up to 100% mutagenesis in some plant lines^30^. We noticed that the sgRNA recognized a sequence in the *PPO* gene with GG just 5′of the PAM sequence, which was shown to promote higher rates of mutagenesis^35^. Furthermore, the Cas9-PPO construct we used is rather specific for DSB induction in the endogenous *PPO* gene, leaving the repair template mostly intact. Therefore, this nuclease was used for DSB induction for *in planta* GT experiments.

Nuclease-induced DSBs may be repaired by end-joining possibly resulting in mutations. Such mutated target is no longer available for DSB induction and for repair with the repair template. This may happen in LSL-PPO/Cas9-PPO plant lines. However, by crossing the LSL-PPO/Cas9-PPO lines with REP plant lines, offspring will contain at least one intact *PPO* gene, since the REP plant line will have two intact endogenous *PPO* alleles. After crossing we tested about 200 F1 seeds on butafenacil, but no seeds showed resistance. Expression of the Cas9-PPO nuclease immediately after fertilization and during embryo formation, was apparently not sufficient to generate GT events that were passed to all the cells of the seedling. The GT events we obtained were found in the next generation and must have been transmitted from the F1 generation via germ line cells. This means that the GT events must have occurred in specific cells i.e. the L2 layer of meristems. However, DSB repair in somatic cells is mainly via NHEJ or HR with the sister chromatid, and the majority of the DSBs will in the end not result in GT events but in mutations. Equipping the nuclease genes with germ-line specific promoters might increase the number of heritable GT events as was shown for DSB induced mutations^36^.

Homologous recombination with repair templates pre-inserted elsewhere in the genome without further processing like replication or excision simplifies the *in planta* GT method. The only essential step is the induction of a DSB in the target locus by an efficient site-specific nuclease. Selection or high through-put identification methods for detection of GT may be required, but the use of germ line promoters for expression of the nuclease might simplify this step and further increase the efficiency of *in planta* GT.

## Methods

### Vector construction and plant transformation

Cas9-PPO (pSDM3905) was described previously^30^.

The LSL region of pLSL^26^ was cloned as *Sda*I fragment in *Sda*I digested pCambia2300. The *Sal*I-*Sma*I fragment of pSDM3900^5^ containing the PPO repair template with two mutations for butafenacil resistance (S305L and Y426M) and a *Kpn*I site at position E445/A446 was cloned in *Sal*I-*Eco*RV digested pEntry4. The LSL-PPO GT construct was created by a Gateway LR reaction, resulting in pSDM3904.

For cloning Cas9-PPO and the PPO repair template on one T-DNA, Cas9 and the gRNA were excised from Cas9-PPO (pSDM3905) by using *Nhe*I, blunted with Klenow, digested with *Bam*HI and ligated into *Mls*I/*Bam*HI linearized pDONR-PPO, which contained the PPO repair template. The fragment containing Cas9-PPO and the PPO repair template of the resulting vector was inserted between the LSL repeats of pMDC100-LSL by a Gateway LR reaction, resulting in pSDM3910. Full details of the plasmids are available upon request.

Plant vectors were introduced in *Agrobacterium tumefaciens* AGL1^37^ by electroporation. Arabidopsis plants (ecotype Col-0) were transformed via the floral dip method^38^ and T1 seeds were selected on MA solid medium without sucrose supplemented with nystatin (100 µg/ml), timentin (100 µg/ml), kanamycin (30 µg/ml) for selection of LSL-PPO or LSL-PPO-Cas9-PPO T-DNA, phosphinothricin (15 µg/ml) for selection of Cas9-PPO T-DNA. Arabidopsis plants transformed with *Agrobacterium* strain GV3101/pMP90/pREP^26^ carrying the 35S-*REP* gene were grown on 1/2 MS with 10 µg/ml hygromycin. GT events were selected on MA solid medium with 0.5% sucrose supplemented with nystatin (100 µg/ml), timentin (100 µg/ml), 50 nM butafenacil.

### DNA isolation and PCR analysis

Seedlings, leaves or flowers were disrupted to a powder under liquid N_2_ in a TissueLyser (Retch, Haan, Germany). DNA was isolated as described^4^. One µl (usually 0.1 µg DNA) was used for PCR reactions in a final volume of 25 µl with Phusion (Finnzymes, Espoo, Finland) or Dreamtaq (Thermo Scientific) polymerase. PCR primers are shown in Table S2. For footprint analysis, the Cas9-PPO nuclease target region of the *PPO* gene was amplified using primers SP392 and SP538. Loss of the *Fau*I restriction site was used for detection of footprints. For detection of GT events the primers PPO-PA (sense) and SP319 (antisense) were used, followed by digestion of the PCR products with *Kpn*I. For detection of Cas9-PPO, LSL-PPO or *Rep* primers were used as shown (Table S2)

### Southern blot analysis

Plant DNA (10 µg) was digested with *Kpn*I and *Xho*I and separated in 0.7% agarose gels, blotted onto Hybond-N and hybridized at 42 °C in DIG Easy Hyb (Roche Diagnostics, Mannheim, Germany) supplemented with 50 µg/ml herring sperm DNA with 50 ng/ml 5′*PPO* probe (primers SP574 and SP575) or 3′*PPO* probe (primers SP576 and SP577), labelled in a PCR reaction using DIG-labelling mix (Roche Diagnostics, Mannheim, Germany). After 16–20 hr, blots were washed twice with 2xSSC; 0.1% SDS at room temperature and three times with 0.2xSSC; 0.1% SDS at 65 °C. Detection was performed using the DIG Wash and Block Buffer set and CDP-Star (Roche Diagnostics, Mannheim, Germany) according to the manufacturers protocol.

### Data availablity

The datasets and materials generated during and/or analysed during the current study are available from the corresponding author on request.

## Electronic supplementary material

Supplementary information
