# Supplementary material for: True gene-targeting events by CRISPR/Cas-induced DSB repair of the PPO locus with an ectopically integrated repair template
Source: Sci Rep. 2018 Feb 20;8:3338. doi: 10.1038/s41598-018-21697-z (PMC5820285; doi:10.1038/s41598-018-21697-z)
Supplement: Supplementary file 1 — Supplementary information [file 41598_2018_21697_MOESM1_ESM.pdf]

True gene-targeting events by CRISPR/Cas-induced DSB repair of the PPO locus with an ectopically integrated repair template

Sylvia de Pater*, Bart J.P.M. Klemann and Paul J.J. Hooykaas

Department of Molecular and Developmental Genetics, Institute of Biology, Leiden University, Leiden, 2333 BE, The Netherlands.

*Corresponding author: Sylvia de Pater b.s.de.pater@biology.leidenuniv.nl

Supporting information

Figure S1: Southern blot detecting the wild type PPO locus, the LSL-PPO repair template and the GT PPO locus.
Figure S2: PCR analysis for the presence of LSL-PPO, Cas9-PPO and Rep in progeny plants.
Figure S3: Quantification of PPO sequences by qPCR.
Figure S4: Full-size gels used to create Fig. 2B.
Figure S5. Full-size gels used to create Fig. 3B.
Figure S6. Full size blots used to create Fig. 3C.
Figure S7. Full-size gels used to create Fig. 4A.
Figure S8. Full-size gels used to create Fig. 4B.
Figure S9. Full-size blots used to create Fig. 5.
Figure S10. Full-size blots used to create Fig. S1B.
Figure S11. Full-size gels used to create Fig. S2.
Table S1. Plant lines and corresponding number of seeds germinated on butafenacil and GT events obtained.
Table S2. Primers used for cloning and PCR reactions.
Table S3. Butafenacil resistance of germinated seeds of indicated plant lines.

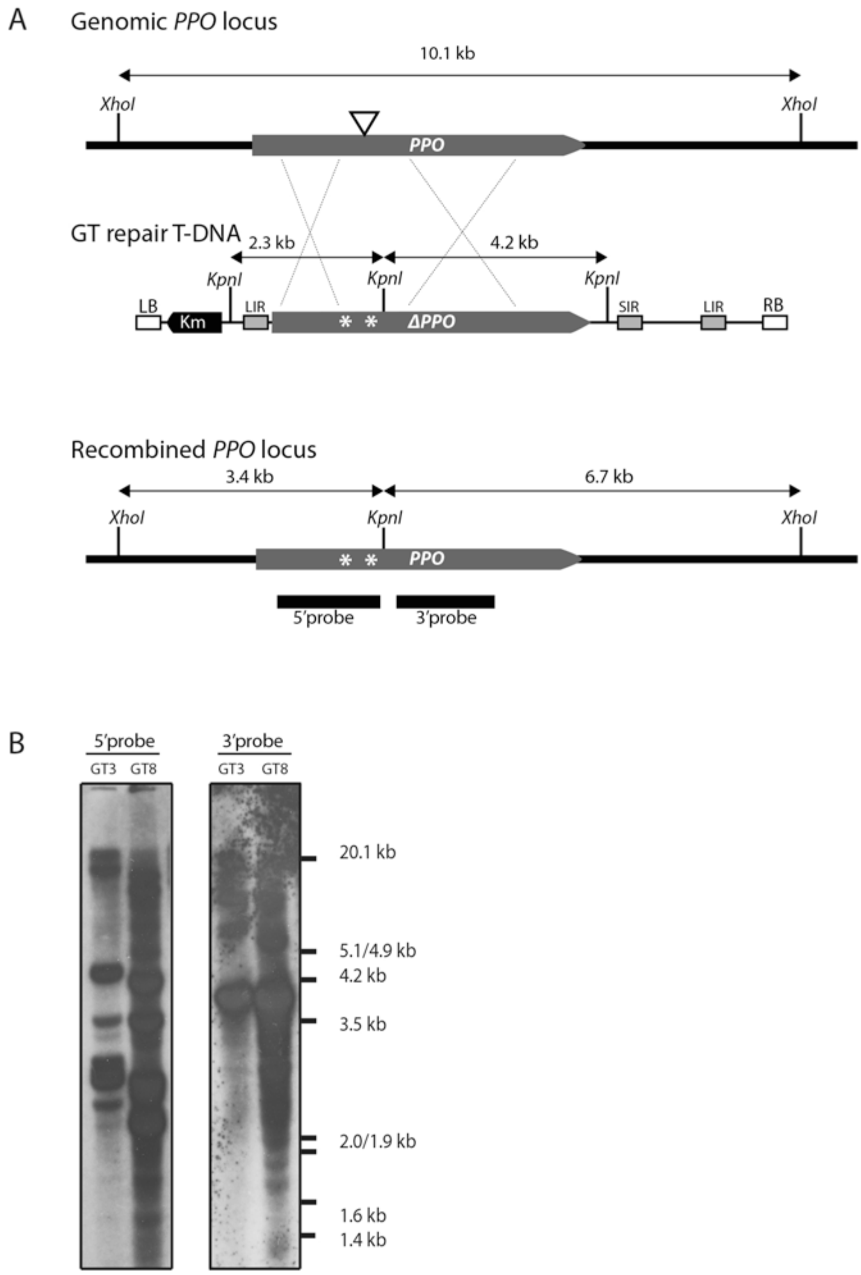

Figure S1. Southern blot analysis detecting the wild-type *PPO* locus, the T-DNA LSL-PPO repair template and the recombined *PPO* locus. A. The coding region of the *PPO* gene is shown as a grey bar; the Cas9 target site in the *PPO* gene as a triangle. The GT repair construct, missing the 5' region of the *PPO* gene including the first 364 bps of the coding sequence, contains base pair substitutions leading to two amino acids changes (indicated by asterisks) (S305L and Y426M) causing insensitivity for the herbicide butafenacil and a *Kpn*I site for detection of GT events. The truncated *PPO* gene is surrounded by LIR and SIR sequences for rolling circle replication by the REP protein. The kanamycin gene on the GT repair construct is used for selection of transformants. Sizes of DNA fragments expected after digestion with selected restriction enzymes are indicated. Probes used for Southern blotting are shown as black bars. B. Southern blot analysis of GT3R-1 and GT8R-1. Ten µg DNA were digested with *Kpn*I and *Xho*I, separated on 0.7% agarose gels and hybridized with a 5'*PPO* probe or 3'*PPO* probe. The sizes of DIG-labelled Lambda *Eco*RI/*Hind*III marker bands are shown. The full-size blots are presented in Fig. S10.

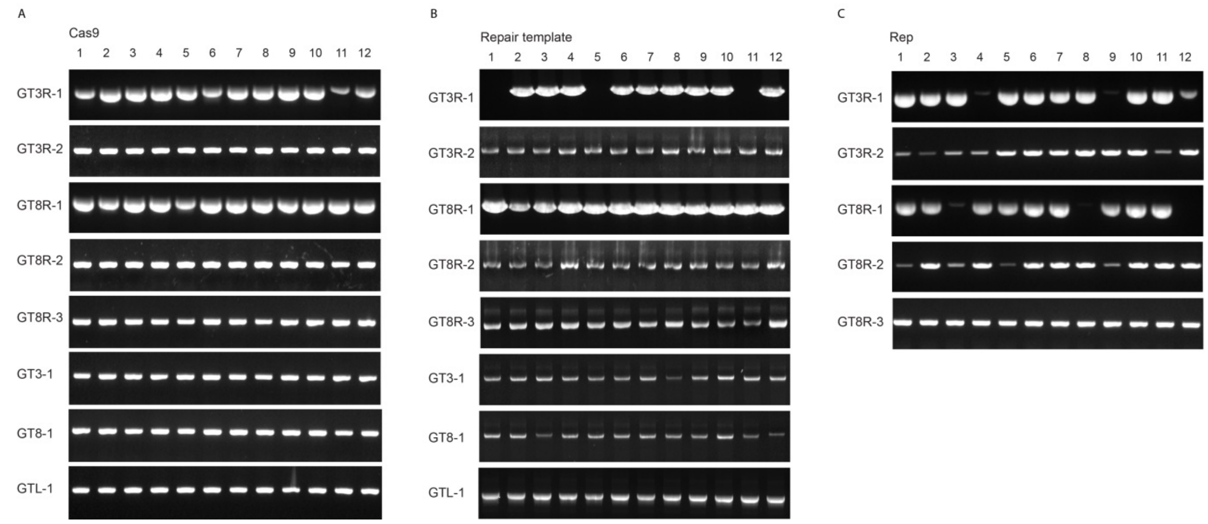

Figure S2. PCR analysis of 12 progeny plants of GT plant lines (GT3R-1, GT3R-2, GT8R-1, GT8R-2, GT8R-3, GT3-1, GT8-1 and GTL-1) for the presence of Cas9-PPO nuclease (A), LSL-PPO repair template (B) and Rep (C). PCR was performed with primers shown in Table S1. The full-size gels are presented in Fig. S11.

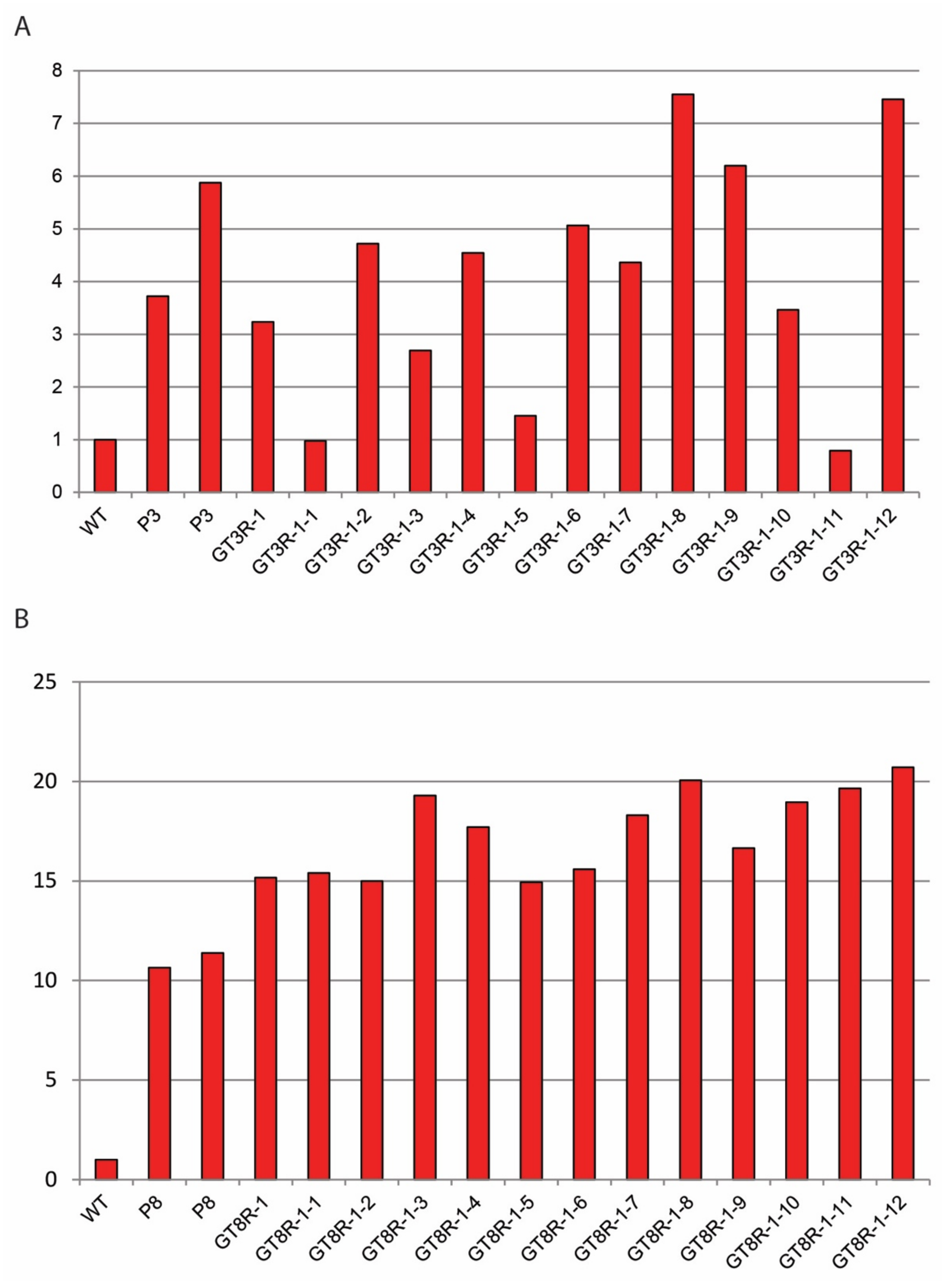

Figure S3. Quantification of PPO sequences by qPCR using DNA from (A) wild type, P3, GT3R-1 and 12 progeny plants of GT3R-1 and (B) wild type, P8, GT8R-1 and 12 progeny plants from GT8R-1. PPO sequences were normalized to sequences of the housekeeping gene *ROC1* (At4g38740). PPO sequences were amplified with SP578 (GGATACCGTAAAGCACGAGG) and SP579 (TCTGGACGAAGAGCATCAGG) and ROC sequences with ROC3.3 (CCACAGGCTTCGTCGGCTTTC) and ROC5.2 (GAACGGAACAGGCGGTGAGTC). Ratio PPO /ROC is shown. Wild type is set at 1.

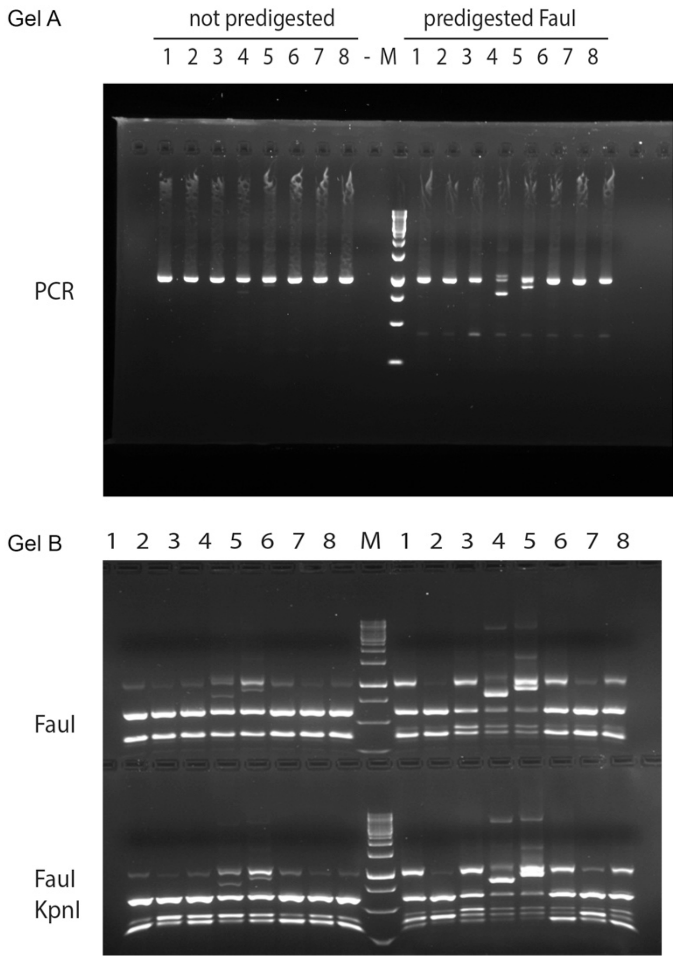

Figure S4. Full-size gels used to create Fig.2B. Gel A has been used for panel PCR and gel B for panels FauI and FauIKpnI. Footprint analysis of plant lines P1-P8. Genomic DNA was pre-digested with *Fau*I or not pre-digested and the *PPO* target site was amplified and an aliquot was separated on a 1.5% agarose gel. The PCR products were digested with *Fau*I or a combination of *Fau*I and *Kpn*I and separated on 1.5% agarose gels. A negative control not containing template DNA (-) was included in the PCR reaction. M is the 1 kb DNA marker.

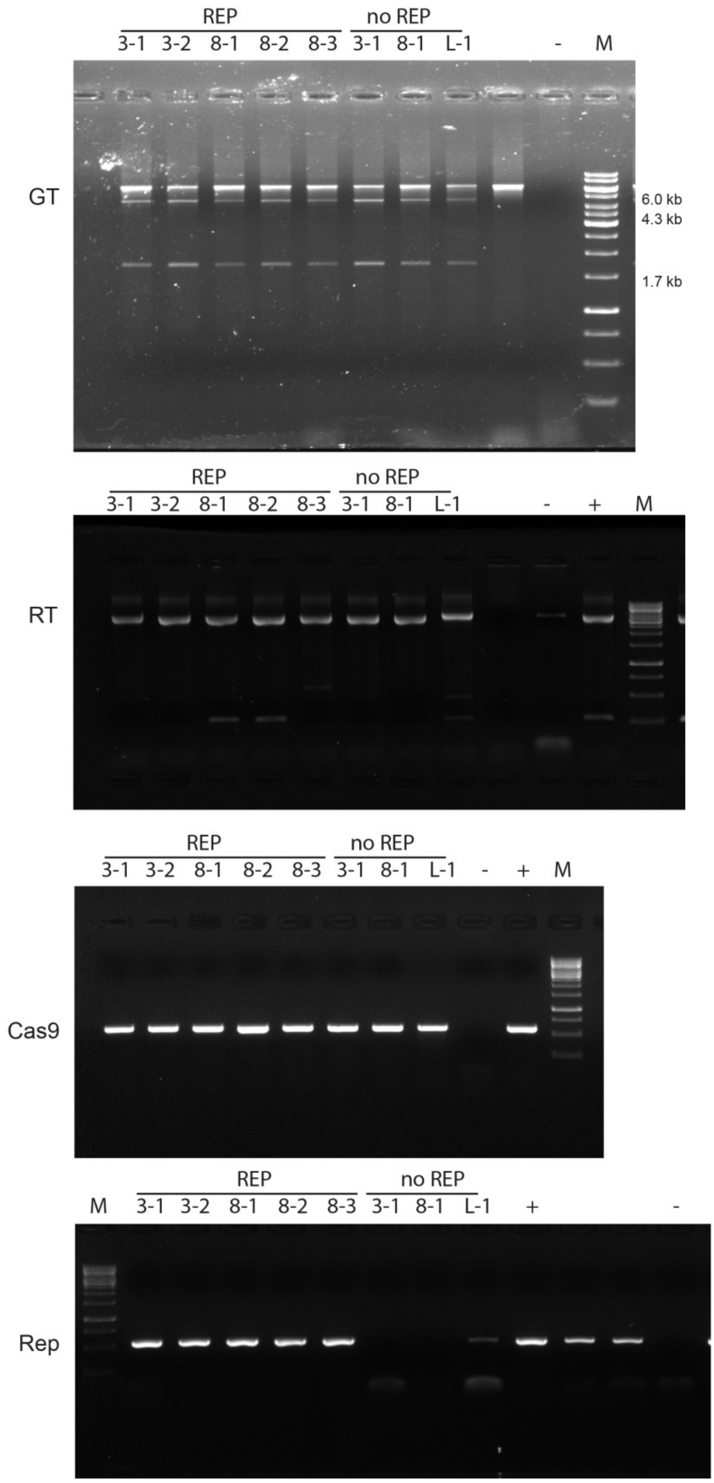

Figure S5. Full-size gels used to create Fig. 3B. GT lines (GT3R-1, GT3R-2, GT8R-1, GT8R-2, GT8R-3, GT3-1, GT8-1 and GTL-1) were tested for the presence of GT, LSL-PPO repair template (RT), Cas9-PPO and REP by PCR. GT products were detected with primer pair PA-SP319. PCR products were digested with *Kpn*I. Sizes of bands after KpnI digestion are indicated. M is the 1 kb DNA marker. Negative controls not containing template DNA (-) and positive controls (+) were included in the PCR reaction.

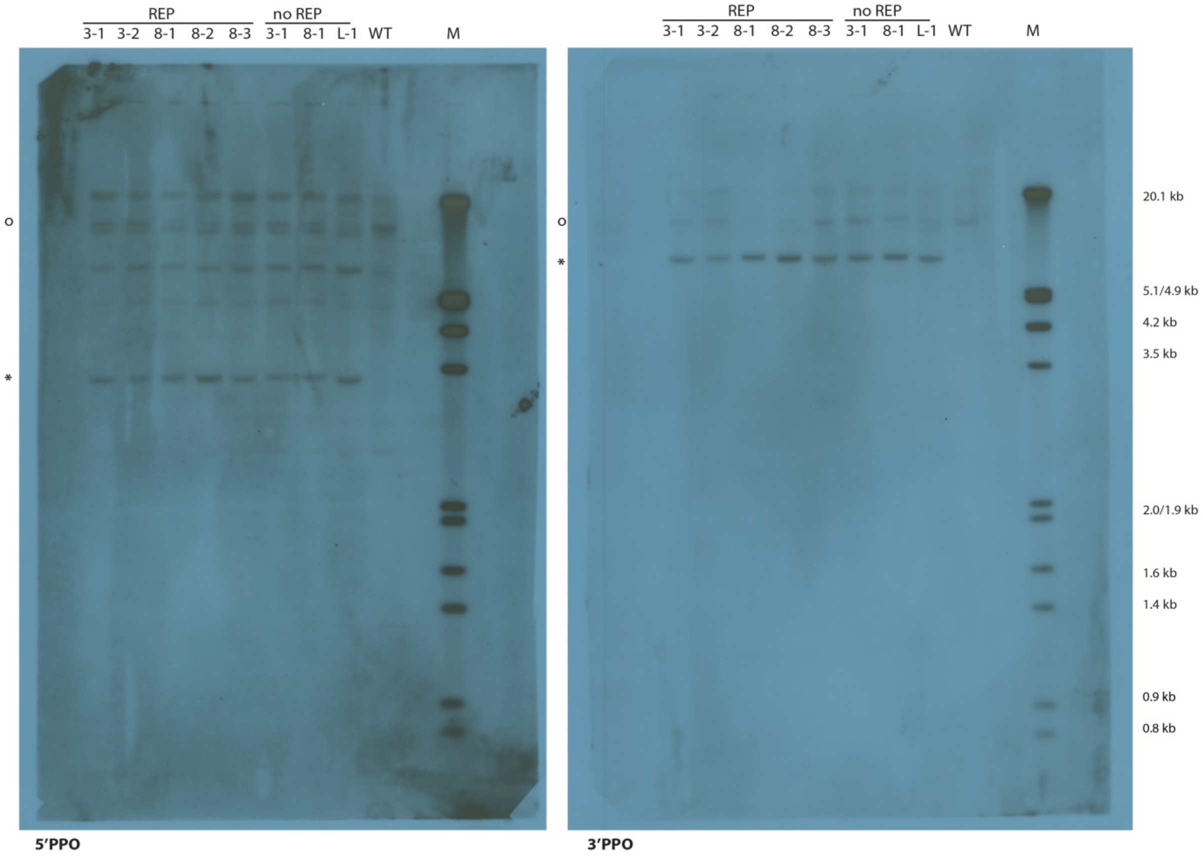

Figure S6. Full size blots used to create Fig. 3C. Southern blot analysis of GT lines. Ten µg DNA of wild-type plants (WT) or GT lines (GT3R-1, GT3R-2, GT8R-1, GT8R-2, GT8R-3, GT3-1, GT8-1 and GTL-1) were digested with *Kpn*I and *Xho*I, separated on 0.7% agarose gels and hybridized with a 5'*PPO* probe (left panel) or 3'*PPO* probe (right panel). The circles indicate wild-type bands (10.1 kb). Asterisks indicate bands with the expected sizes (3.4 kb and 6.7 kb) after GT by HR. Lanes M contain DIG-labelled Lambda *Eco*RI/*Hind*III marker. The sizes of the marker bands are shown.

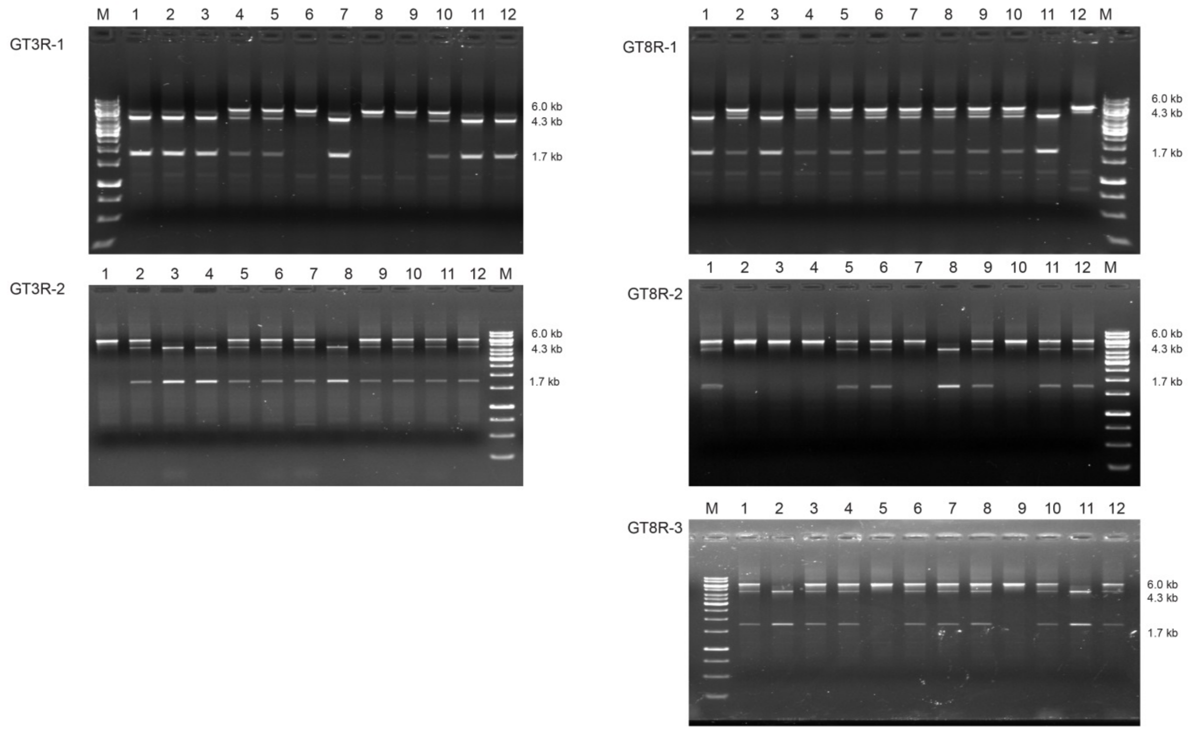

Figure S7. Full-size gels used to create Fig. 4A. Twelve progeny plants of GT3R-1, GT3R-2, GT8R-1, GT8R-2 and GT8R-3 were tested for the presence the wild-type *PPO* locus (6.0 kb product) or GT *PPO* locus (4.3 kb and 1.7 kb products) by PCR with primers PA-SP319 and subsequent *Kpn*I digestion. M is the 1 kb DNA marker.

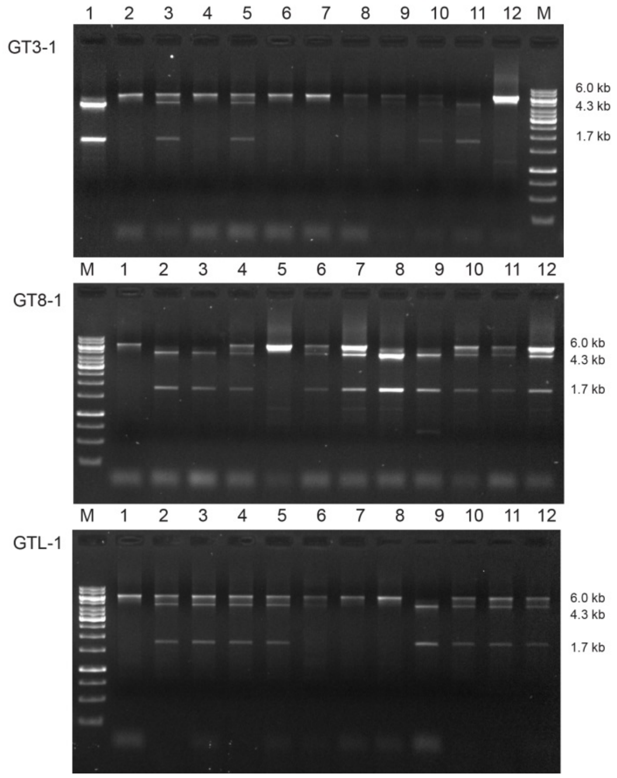

Figure S8. Full-size gels used to create Fig. 4B. Twelve progeny plants of GT3-1, GT8-1 and GTL-1 were tested for the presence the wild-type *PPO* locus (6.0 kb product) or GT *PPO* locus (4.3 kb and 1.7 kb products) by PCR with primers PA-SP319 and subsequent *Kpn*I digestion. M is the 1 kb DNA marker.

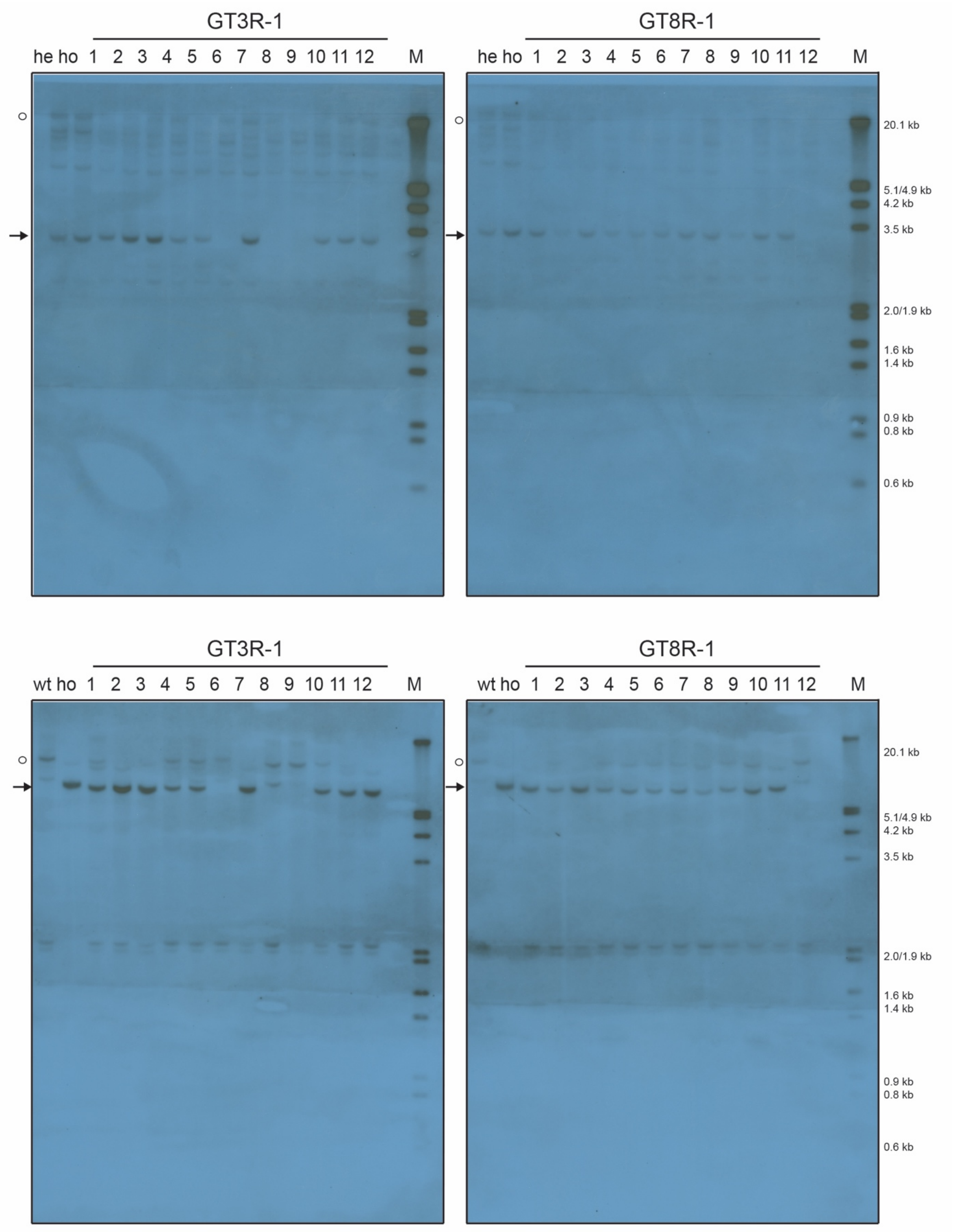

Figure S9. Full-size blots used to create Fig. 5. Southern blot analysis of progeny of GT3R-1 and GT8R-1. Ten µg DNA of wild-type plants (WT), control heterozygous GT plants (he), control homozygous GT plants (ho), GT3-1 and GT8-1 progeny (1-12) were digested with *Kpn*I and *Xho*I, separated on 0.7% agarose gels and hybridized with a 5'*PPO* probe (upper panels) or 3'*PPO* probe (lower panels). The dots indicate wild-type bands (10.1 kb). Arrows indicate bands with the expected sizes (3.4 kb and 6.7 kb) after GT by HR. Lanes M contain DIG-labelled Lambda *Eco*RI/*Hind*III marker. The sizes of the marker bands are shown.

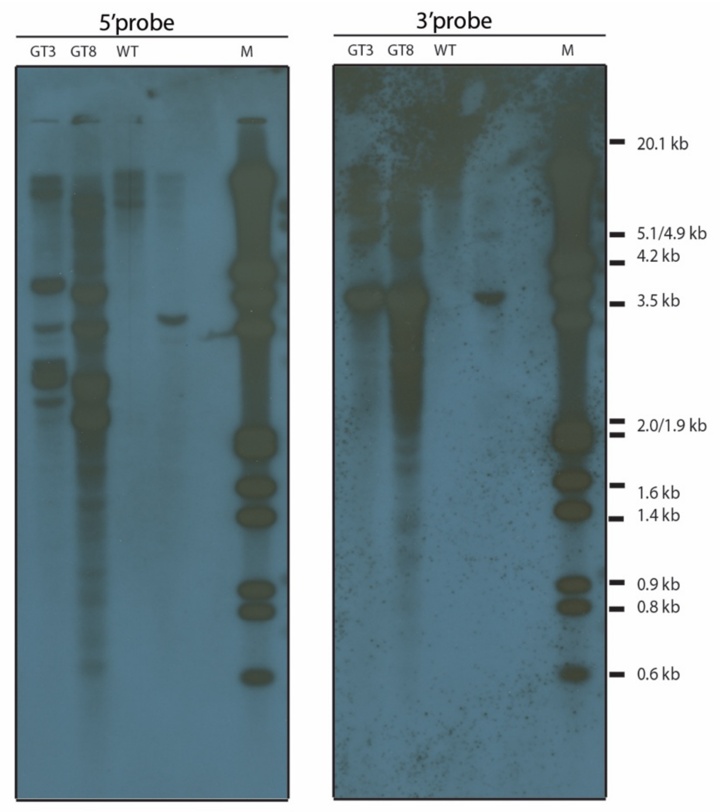

Figure S10. Full-size blots used to create Fig. S1B. Southern blot analysis of GT3R-1, GT8R-1 and wild type (WT). Ten µg DNA were digested with *Kpn*I and *Xho*I, separated on 0.7% agarose gels and hybridized with a 5’*PPO* probe or 3’*PPO* probe. The sizes of DIG-labelled Lambda *Eco*RI/*Hind*III marker bands are shown.

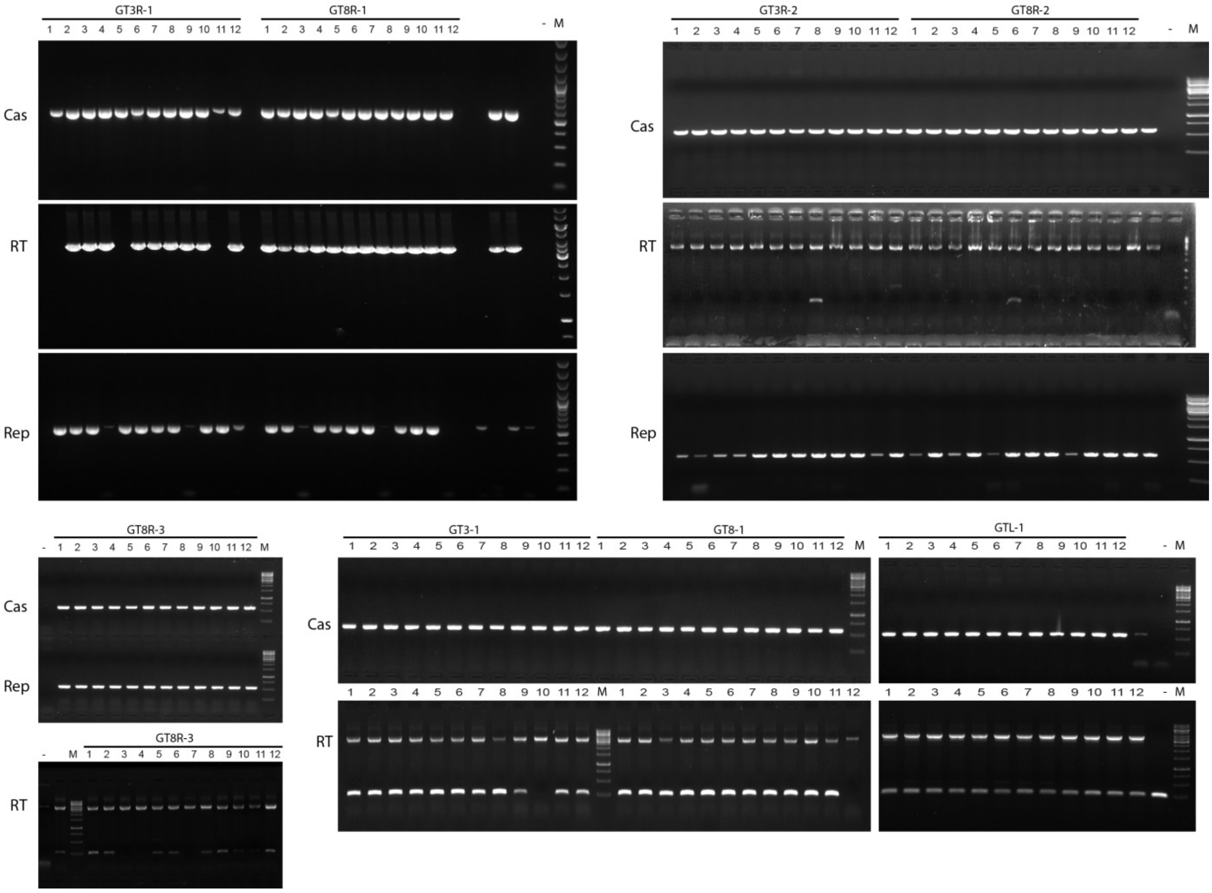

Figure S11. Full-size gels used to create Fig. S2. PCR analysis of 12 progeny plants of GT plant lines (GT3R-1, GT3R-2, GT8R-1, GT8R-2, GT8R-3, GT3-1, GT8-1 and GTL-1) for the presence of Cas9-PPO nuclease (Cas), LSL-PPO repair template (RT) and Rep. PCR was performed with primers shown in Table S1. M is the 1 kb DNA marker except for panels GT3R-1/GT8R-1 Cas and Rep where the 100 bp DNA marker was used. Negative controls not containing template DNA (-) were included in the PCR reaction.

Table S1. Plant lines and corresponding number of seeds germinated on butafenacil and GT events obtained.

Plant lines	Seeds germinated on butafenacil	GT events
P2 x Rep119	7500	
P3 x Rep119	50000	1
P4 x Rep119	7500	
P5 x Rep119	7500	
P8 x Rep119	11250	
P3 x Rep124	41500	1
P4 x Rep124	8750	
P5 x Rep124	7500	
P8 x Rep124	66000	3
P3	50000	1
P8	50000	1
PL1	6000	1
PL2	12000	
PL3	12000	

Table S2. Primers used for cloning and PCR reactions.

Primer	Sequence	Used for
SP392	CACTTTGACAGATTAGGTAG	Footprint analysis PPO
SP538	CTAAGGCTACACCAGCGACG	Footprint analysis PPO
PPO-PA	GTGACCGAGGCTAAGGATCGT	GT detection
SP319	CTATCAAAGAGCACAGACAGC	GT detection
SP558	GGAACTAACTCTGTGGGATG	Cas9 detection
SP559	CACACCTGAAGCGTTGATAG	Cas9 detection
SP157	GTCCCATTCAACTATCTTGGTAAG	LSL-PPO detection
SP519	CGGAAGCTTTAGCAGAAGGCATGTTGTTGTG	LSL-PPO detection
SP536	GCCATGGAGTGAATGCCTTCTGCTAGTAAGAAC	Rep detection
SP548	CTGATTCTGTGAAGGGCGAC	Rep detection
PPO-4	CATGAAGTTGTTGACCTCAATC	Sequencing of GT events
SP574	CTAGTAATGTATAAGTGGTAATCATC	Southern blot 5’ probe
SP575	GGTTTAGAGTTGGGTTTGACC	Southern blot 5’ probe
SP576	GCTGTCTGTGCTCTTTGATAG	Southern blot 3’ probe
SP577	GCTGCTCTTGTTTCTTCTTGTAC	Southern blot 3’ probe

Table S3. Butafenacil resistance of germinated seeds of indicated plant lines. Number of resistant (But^R) and sensitive (But^S) seedlings is shown.

Plant line	But^R	But^S	% But^R
P3 (T2)	0	100	0
P8 (T2)	0	100	0
GT3 (T2)	70	24	74
GT8 (T2)	94	13	88
WT	0	100	0
GT3-1 (T3)	99	0	100
GT3-2 (T3)	100	0	100
GT3-3 (T3)	100	0	100
GT3-4 (T3)	69	29	70
GT3-5 (T3)	72	27	73
GT3-6 (T3)	0	100	0
GT3-7 (T3)	100	0	100
GT3-8 (T3)	0	100	0
GT3-9 (T3)	0	100	0
GT3-10 (T3)	70	28	71
GT3-11 (T3)	100	0	100
GT3-12 (T3)	100	0	100
GT8-1 (T3)	100	0	100
GT8-2 (T3)	73	26	74
GT8-3 (T3)	98	0	100
GT8-4 (T3)	82	17	83
GT8-5 (T3)	74	22	77
GT8-6 (T3)	72	27	73
GT8-7 (T3)	65	34	66
GT8-8 (T3)	78	21	79
GT8-9 (T3)	81	18	82
GT8-10 (T3)	69	31	69
GT8-11 (T3)	98	0	100
GT8-12 (T3)	0	100	0
